# Equine Coital Exanthema: New Insights on the Knowledge and Leading Perspectives for Treatment and Prevention

**DOI:** 10.3390/pathogens10081055

**Published:** 2021-08-20

**Authors:** María Aldana Vissani, Armando Mario Damiani, María Edith Barrandeguy

**Affiliations:** 1 Instituto de Virología CICVyA, Instituto Nacional de Tecnología Agropecuaria (INTA), Dr. Nicolás Repetto y De Los Reseros s/nº, Hurlingham B1686LQF, Argentina; barrandeguy.maria@inta.gob.ar; 2 Cátedra de Enfermedades Infecciosas, Escuela de Veterinaria, Facultad de Ciencias Agrarias y Veterinarias, Universidad del Salvador, Champagnat 1599, Ruta Panamericana km 54.5, Pilar B1630AHU, Argentina; 3 Consejo Nacional de Investigaciones Científicas y Técnicas (CONICET), Godoy Cruz 2290, Argentina; amdamiani9@gmail.com; 4 Instituto de Medicina y Biología Experimental de Cuyo IMBECU, Área de Química Biológica, Facultad de Ciencias Médicas, Universidad Nacional de Cuyo, Mendoza M5500, Argentina

**Keywords:** equid alphaherpesvirus, equine coital exanthema, EHV-3, equine industry, equine reproduction, equine infectious disease

## Abstract

Equine coital exanthema (ECE) is a highly contagious, venereally-transmitted mucocutaneous disease, characterized by the formation of papules, vesicles, pustules and ulcers on the external genital organs of mares and stallions, and caused by *equid alphaherpesvirus 3* (EHV-3). The infection is endemic worldwide and the virus is transmitted mainly through direct contact during sexual intercourse and by contaminated instruments during reproductive maneuvers in breeding facilities. The disease does not result in systemic illness, infertility or abortion, yet it does have a negative impact on the equine industry as it forces the temporary withdrawal of affected animals with the consequent disruption of mating activities in breeding facilities. The purpose of this review is to provide up-to-date relevant information on the knowledge of EHV-3 infection and to analyze new approaches on diagnostics, treatment and prevention in the interest of minimizing the negative consequences of ECE in light of the current situation of the equine industry.

## 1. Introduction

Equine coital exanthema (ECE) is a highly contagious, venereally-transmitted mucocutaneous disease, characterized by the formation of papules, vesicles, pustules and ulcers on the external genital organs of mares and stallions, and caused by *equid alphaherpesvirus 3* (EHV-3) [1,2].

The disease, firstly described in Ireland in the early 1900s [3], was then concurrently isolated during the 1960s in the United States, Canada and Australia [4,5,6]. Since then, ECE, known also as genital horse pox, eruptive venereal disease, equine venereal vulvitis or balanitis, or coital vesicular exanthema, has been reported almost worldwide [7,8,9,10,11,12,13,14,15,16]. Recently, EHV-3 infection was, for the first time, communicated in the native Icelandic horse population [17].

The infection is relatively benign and does not result in systemic illness, infertility or abortion [11,18]. However, the negative impact on the equine industry relies on the forced, temporary withdrawal of affected animals with the consequent disruption of mating activities in breeding facilities [1,2,19].

The purpose of this review is to provide up-to-date relevant information on the knowledge on EHV-3 infection and to analyze new approaches on diagnostics, treatment and prevention in the interest of minimizing the negative consequences of ECE in light of the current situation of the equine industry.

## 2. Classification, Taxonomy, and Structure of EHV-3

Equid herpesviruses (Order Herpesvirales, family *Herpesviridae*) are classified in two subfamilies, named *Alphaherpesvirinae* and *Gammaherpesvirinae.* EHV-3 is a member of the *Alphaherpesvirinae* subfamily within the genus *Varicellovirus*, which includes also other equine herpesviruses: *equid alphaherpesvirus 1* (EHV-1), *equid alphaherpesvirus 4* (EHV-4), *equid alphaherpesvirus 6* (EHV-6), *equid alphaherpesvirus 8* (EHV-8) and *equid alphaherpesvirus 9* (EHV-9) [20,21,22,23]. As to EHV-3, it has a double-stranded DNA class D genome consisting of a long and a short unique region (UL and US), both flanked by inverted repeats (TRL/IRL and IRS/TRS) [24]. The only available EHV-3 complete genome sequence consists of 151,601 nt (G + C content of 68.1%) codifying for 76 genes, four of which are duplicated (ORFs 64, 65, 66, and 76), resulting in a total of 80 genes [25]. Based on the nucleotide sequence of the equine alphaherpesviruses, EHV-3 is the most divergent one, with overall nucleotide identities ranging from 62.1% to 64.9%, whereas EHV-1, EHV-4, EHV-8, and EHV-9 have identities of at least 78.2% [25]. As to EHV-6, the counterpart of EHV-3 in donkeys (also referred to as asinine herpesvirus 1; AsHV-1), it was recently identified as genetically closely related to EHV-3 on the basis of phylogenetic analysis of the viral glycoproteins (g) B, C, and D, resulting in an overall nucleotide sequence identity of 87.02% [26]. Then, AsHV-1 falls together with EHV-3 within the same clade, distinct from the clade formed by the other equine herpesviruses mentioned before.

Antigenic variants of EHV-3 have not been described yet, and there is not a detailed analysis of the antigenic and immunogenic proteins/glycoproteins of EHV-3 [1,2]. However, as stated by Barrandeguy (2010), a genetic analysis of the partial sequence of the gG gene from 25 field isolates demonstrated that there are at least four genetically distinguishable strains in circulation, named CAT, AAT, CAG and ACT. These variants are a consequence of three base substitutions at positions 904, 1103 and 1264 of the gG gene, being the one at position 904 a silent mutation, while those at 1103 and 1264 produce changes in the amino acid sequence: aspartic acid to alanine in the amino acid position 368, and serine to alanine in the 422 of the gG protein [27]. Unfortunately, the biological implications of this are still undetermined. The recently reported cloning of EHV-3 as a bacterial artificial chromosome (BAC) provides a reliable tool to study EHV-3 genome structure and gene function. Mutant EHV-3 viruses devoid of gE or gG have been shown to be dispensable for virus growth in vitro [28,29]. As for other important equine alphaherpesvirus pathogens [30,31], the simple manipulation of the EHV-3 genome cloned as BAC could significantly contribute to the understanding of EHV-3 replicative cycle and also to the characterization of its protein coding genes in pursuance of better comprehending the pathogenesis.

Like other herpesviruses, EHV-3 has a viral envelope, with specific glycoproteins necessary for viral–host interactions, which must remain intact for transmission and infection. It is extensively known that the infectivity is quickly cleared by lipid solvents, detergents, common disinfectants for veterinary use, heat, drying and ultraviolet radiation [32,33,34]. However, some controversies on this issue have arisen during the last years; studies done with EHVs in fomites have shown variable periods of stability and viability, mainly dependent on the type of surface of contaminated objects, humidity and temperature, and, in the case of water contamination, water properties as pH, salinity, temperature, presence of biological sediments and level of pollution [35,36,37]. The main limitation of these studies is that the results have been obtained under laboratory conditions, which do not completely represent what happens in field conditions.

## 3. Epidemiology and Transmission of EHV-3

**Figure 1:**
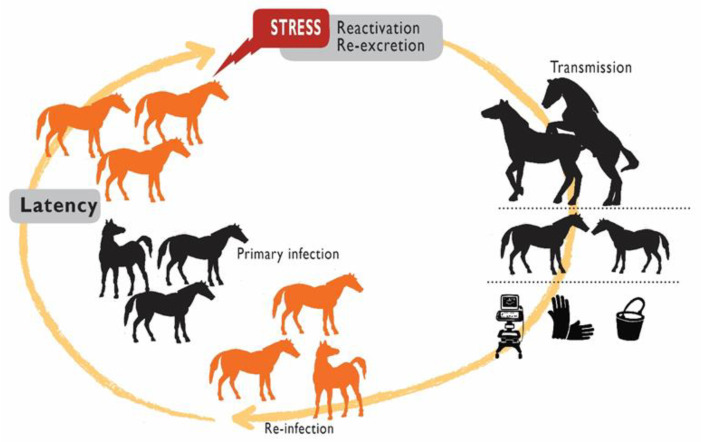
Transmission cycle of *equid alphaherpesvirus 3* (EHV-3) in mares and stallions.

EHV-3 is endemic in most equine breeding populations in the world. The virus is transmitted mainly through direct contact during sexual intercourse [1,2]; nevertheless, non-coital transmission has been occasionally described, associated with the genito–nasal contact by behavioral nuzzling/sniffing [38,39,40]. Contaminated fomites are frequently implicated in the spread of this virus, during reproductive maneuvers in breeding facilities; fomites such as examination gloves, ultrasound scanner, elements used for hygiene of the genital area of mares and stallions or surgical instrument in the post-partum management (Figure 1) [13,41]. The use of a contaminated endoscope was involved in an outbreak of an atypical presentation of EHV-3 infection resulting in unilateral rhinitis in training Thoroughbred horses [12]. Although it has been suggested [16,36], there is neither scientific evidence to support transmission via mechanical vectors, such as stable flies; nor by urine from infected animals. Finally, it has been suggested that the virus can potentially be transmitted to the ejaculate through penis contact with the artificial vagina or sleeves, and consequently by artificial insemination with fresh or frozen semen [36,42]. However, the consequences of this transmission route should be dismissed as there is no information regarding the susceptibility of cylindrical epithelium of endocervix and endometrium to EHV-3 infection.

**Figure 2:**
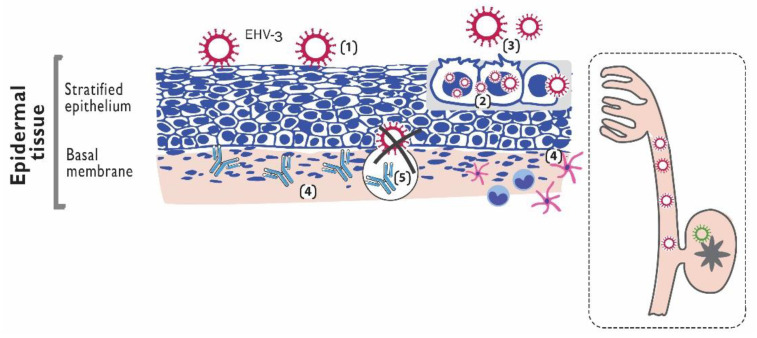
(1) *Equid alphaherpesvirus 3* (EHV-3) infects the stratified epithelium of epidermal tissues present at mucous–cutaneous margins and skin of external genital organs; (2) the virus replicates and laterally spreads; (3) lytic replication occurs and the virus is shed; (4) destruction of epithelial cells elicits a vigorous, localized inflammatory response; (5) the virus does not breach the basal membrane, and thus systemic dissemination is limited; square to dots: After active infection the virus induces latency; the anatomic site has been not yet demonstrated but it has been inferred to be in the sciatic and/or sacral ganglion cells.

Just as for other herpesvirus infections, latency and reactivation are critical events in the epidemiology of EHV-3 [1]. During periods of reactivation from latency, production and excretion of infectious virus serve as the source of infection for other animals (Figure 1) EHV-3 latency has been demonstrated experimentally by reactivation and re-excretion after the administration of glucocorticoids to seropositive mares [43] and also naturally, by isolation of the virus from seropositive mares that remained isolated from other equids during 11 months [44]. Although the anatomic site for latency has been inferred to be in the sciatic and/or sacral ganglion cells based on comparison with *human herpesvirus 2* and *caprine alphaherpesvirus 1* (CpHV-1) [1], it has not yet been demonstrated experimentally (Figure 2).

Episodes of reactivation and re-excretion, either with or without clinical manifestation, and together with variable levels of serum antibodies, have been reported, with the horses undergoing such episodes being the source of virus for other animals [1,44,45].

Studies on seroprevalence have shown variable results; certainly, the highest seroprevalence is expected in broodmares and stallions, rather than in sport or working horses, since reproduction is a crucial factor in the epidemiology of EHV-3 infection. A study performed in a breeding facility in Argentina showed EHV-3 antibodies in 48% (105) for 220 broodmares. Similarly, in Turkey, the highest seroprevalence for EHV-3 was detected in breeding stocks (51.2%), followed by racehorses (10.2%) and working horses (9.3%), with higher rates in mares than in stallions [46]. Opposed to these awaited results for sexual mature horses, a study conducted in Japan reported only 4.1% (13/319) of seropositive animals, with higher prevalence in stallions (11.5%) than in mares (2.3%) [47].

## 4. Pathogenesis and Disease Manifestations

After initial infection, EHV-3 replicates in the stratified epithelium of epidermal tissues present at mucous–cutaneous margins and skin of external genital organs of mares and stallions. EHV-3 replication is restricted to genital and nasal mucosa, being EHV-3 induced plaques significantly bigger in vaginal mucosal ex vivo explants rather than in respiratory ones according to a recent study [48], thus confirming the natural tropism of EHV-3 to genital tissues. Results from this study also indicate that EHV-3 replication is restricted to the epithelium and neither breaches the basal membrane in a direct way nor infects individual monocyte immune cells to invade the lamina propria [48]. Then, contrary to the invasive characteristic of EHV-1, systemic dissemination of EHV-3 is limited, but it is still unknown which factors are involved in this localized replication (Figure 2).

The immunity induced by EHV-3 infection has not been yet studied in detail. Serological responses to this infection include the production of complement-fixing and serum-neutralizing antibodies which reach maximum levels 14 to 21 days after infection. Complement-fixing antibodies decline rapidly and are usually not detectable by the time of 60 days after infection, whereas serum-neutralizing antibody activity is maintained for at least 1 year [43,49,50,51]. There is no available information regarding mucosal immunity, but as reactivation and re-excretion occur even in the presence of higher serum-neutralizing antibody titers [44,45], it would be reasonable to hypothesize that mucosal immunity plays an important role in the control of infection and of re-excretion after reactivation, and also in the severity of clinical lesions.

After replication in the mucosal epithelium, cell-to-cell spread of the virus occurs, and destruction of epithelial cells by lytic virus infection elicits a vigorous, localized inflammatory response that gives rise to the formation of characteristic cutaneous lesions of ECE [2]. Anorectal lymphadenopathy has been also suggested as an additional complication associated to EHV-3 infection. However, it could not yet be elucidated if the enlargement of the anorectal lymph nodes is due to EHV-3 infection itself, to the outcome of the inflammatory process in the region, or to a secondary bacterial infection [1,13].

After an incubation period of five to nine days, small (1–2 mm) raised and reddened papules, which can often go unnoticed, appear on the penis and prepuce of stallions, and on vagina, perineum and, occasionally, on teats of mares. Sequentially, lesions progress to vesicles and pustules, and after epidermal sloughing of the necrotic dome of the pustule, a shallow, raw or encrusted erosion or ulcer appears [1,52]. Recently, during an extended outbreak of ECE in Thoroughbred mares, small “cracks” in the rectal sphincter were reported as the most common clinical sign [41]. Localized inflammation, reddening, congestion and edema of the genital area are commonly observed, along with vulvar discharge, tail switching, frequent urination or arching of the back [2,52]. Lymphadenopathy, constipation, tenesmus and evacuation of firm, dry, mucus-covered feces have been related by Barrandeguy et al., (2013) with lesions around the anus during a severe outbreak of ECE at an artificial insemination center [13]. General signs of infection, such as fever, anorexia or dullness, are rare, but if present they are more severe in stallions than in mares. It is noteworthy that stallions with severe lesions can exhibit discomfort, loss of libido and refusal to mate and copulate [1,2,53,54].

Uncomplicated cases typically resolve within 10 to 14 days without any sequelae, while depigmentation and cutaneous scars can persist longer. Severity and duration of ECE lesions are also influenced by secondary bacterial infections (being *Streptococcus zooepidemicus* the most common) and/or external myiasis [13,45,52,53].

## 5. Latest Approaches for the On-Site Diagnosis

A presumptive diagnosis of EHV-3 infection can be made on the basis of clinical presentation with reasonable certainty, but laboratory confirmation is essential for the implementation of biosecurity and control measures. Moreover, diagnosis of subclinical infections, which is critical to prevent outbreaks, cannot be assessed by clinical inspection [1,52].

Not long ago, diagnosis rested on virus isolation [1,2,55] and detection of EHV-3 DNA by conventional or real time PCR [1,7,9,17,45,47], and, though less useful for practical implementation, the demonstration of seroconversion in paired serum samples from the acute and convalescence period within 15–21 days interval [1]. Recently, a new platform based on the fluorescent probe hydrolysis-based insulated isothermal PCR (iiPCR) technology was reported [56]. This on-site diagnostic tool serves for the rapid and accurate detection of EHV-3 in breeding farms, facilitating the identification of sub-clinically infected mares prior to mating, hence avoiding contagion of the stallion and contributing to prevent huge outbreaks of ECE [56].

Independently of the diagnostic methodology to be implemented (virus isolation, conventional, real time or iiPCR), perineal and genital swabs are the selected clinical samples, which should be collected by firm swabbing or scraping of the edges of fresh, active lesions and maintained in 5–6 mL viral transport medium (containing antibiotics and antimycotics) [1].

## 6. New Insights on Treatment, Prevention and Control

The treatment of stallions showing ECE clinical signs has been mainly based on sexual rest until lesions have healed, together with the administration of broad-spectrum antimicrobials to avoid secondary bacterial infections [1,2,52]. In order to reduce viral replication, the use of nucleoside analogues, such as acyclovir (ACV) and ganciclovir (GCV), has been occasionally evaluated, and both drugs have demonstrated to be effective against EHV-3 in vitro [38,57]. A plaque reduction assay comparing the effective concentration of both compounds, showed that GCV was the most potent in reducing plaque number and size [58]. Similar results were obtained recently by means of an impedance-based cellular assay confirming that GCV has a higher efficiency than ACV to inhibit EHV-3 replication [59]. Valacyclovir (VACV; ACV prodrug) was also evaluated in vitro by a plaque reduction assay compared with ACV, and though both showed the same effectiveness against EHV-3 infection in vitro, when oral VACV (27 mg/kg/eight hours for two days and 18 mg/kg/12 h for eight days) was administered to stallions showing clinical ECE, the treatment did not reduce the severity and the duration of clinical disease [54]. Recently, treatment with an incremented oral dose of VACV (35 mg/kg/eight hours for three days and then 25 mg/kg/12 h for nine days) together with the application of a topical ointment of ACV on the penis of three naturally infected stallions, demonstrated a complete inhibition of EHV-3 replication five and eight days after initiation of the treatments [60]. However, it is difficult to arrive to a conclusion regarding the effectiveness of oral VACV and topical ACV, together or separately, against EHV-3, from the previous studies, since these studies were carried out in the face of natural outbreaks without untreated infected animals as controls, and since the concentration and frequency of application of the ACV ointment was not reported. Besides, a recent and more complete study demonstrated that the use of a topical cream containing GCV 0.01% weight/weight as a therapeutic treatment reduces the duration of clinical lesions and loads of virus shedding in experimentally infected mares as compared with placebo and control animals. The same topical compound was studied as a preventive treatment, applied shortly (4 h and 4/24 h) after the experimental EHV-3 challenge (before the expected appearance of genital lesions) with the aim of mimicking the condition of sub-clinically infected mares observed in the field; no significant differences in the severity of the disease, length of virus shedding, or infectious virus load, were found [45]. Further studies to optimize the therapeutic protocol by increasing GCV concentration and/or improving the dosing regimen are ongoing in naturally infected mares [61].

Taking into account that the most important negative consequence of ECE is the occurrence of the disease in stallions during the breeding season, the preventive measures rely mainly on clinical examination of mares before mating, segregating those with clinical evidence of ECE. However, this procedure does not identify sub-clinically infected animals. A study carried out in a breeding farm in Argentina showed that 48% of the mares were seropositive, thus latently infected, and 6% were excreting the virus without clinical signs [44], leading to a high risk of contagion for stallions. Thus, in breeding farms with heavily-scheduled calendars, it is highly recommended to perform strict clinical examination before breeding, and to implement an accurate biosecurity management protocol during the pre-mating hygienic procedures in mares and post-mating in stallions. Moreover, to overcome the possibility that sub-clinically infected mares transmit the virus to stallions, the adoption of additional preventive measures, as identification of these mares by on-site real time PCR and segregation from mating, is strongly recommended [45]. Recently, in an outbreak of ECE occurred in a Thoroughbred farm in Argentina, rectifying management measures and timely implementation of EHV-3 real-time PCR identification resulted critical to minimize the extension of the outbreak and to avoid infection of stallions [41].

Commercial vaccines against ECE are not available, and this preventive alternative has not been yet explored [1,52]. Promising results in prevention have been shown for a genital infection produced by CpHV-1, using vaginal immunization of goats with a vaccine containing the inactivated virus and a mutant enterotoxin of *Escherichia coli*, as adjuvant. Vaccinated goats displayed high levels of secretory IgA and were significantly protected after challenge with the virulent CpHV-1 strain, with pronounced decrease in virus shedding [62,63]. Considering that the infection in goats with CpHV-1 results in similar clinical manifestation than ECE in horses, these findings are auspicious and suggest that local immunization could be a potential alternative for prevention of ECE. In turn, the ability to efficiently manipulate the EHV-3 genome [28] provides a reliable tool for the construction of attenuated strains with vaccine potential. 

## 7. ECE Negative Impact on Horse Industry

As to Thoroughbred, the top-four producers are the United States, Australia, Ireland and Argentina [64]. Since the Thoroughbred industry allows only natural mating, shuttle stallions (around 100/year), fulfill the breeding season in in both the northern and southern hemispheres during the same calendar year [65,66]. Thus, in intensively managed stud operations, heavily scheduled breeding dates are programmed for stallions. Particularly for the Thoroughbred industry, then, the main negative consequence of ECE is the forced and temporary disruption of mating activities of affected stallions. The possibility of iatrogenic EHV-3 dissemination and outbreaks of ECE in mares is another negative consequence, principally in artificial insemination and embryo transfer facilities [1,19].

In accordance with Barrandeguy and Thiry (2012), temporary disruptions in mating activities in Thoroughbred, are translated into significant end-of-season decreases in the number of entries into the mare book of the affected stallions, and delays in foaling dates or reductions in pregnancy rates in mares that miss breeding opportunities due to the disease [1,2,19]. Similarly, in artificial insemination and embryo transfer centers, affected mares become reluctant to be inspected, inseminated or transferred, with the consequent missed chance of pregnancy. Moreover, additional time and necessary precautions required to manage the mares also have a substantial negative impact [1].

## 8. Conclusions

Outbreaks of ECE continue to be a major problem in breeding enterprises during reproductive seasons, as demonstrated by recent communications, because the infection of stallions and mares and consequently the temporal disruption of mattings, have an economically important impact on the equine industry.

In the interest of reducing the negative impact of EHV-3 infections on the breeding industry, advances had been made during the last years as to diagnostic methodologies, treatment and prevention. The new technology for on-site diagnosis, which is currently available for use, allows rapid and accurate identification of sub-clinically infected mares before mating, being also a tool for the confirmation of clinical observations during the inspection. Concerning antiviral treatment, although there is still not a commercial product available for use, approaches have been performed, with promising results, with a topical GCV treatment; its application contributes to the clearance of virus shedding, thus reducing the time mares and stallions are segregated from reproduction.

Therefore, in a heavily scheduled breeding facility, the combined implementation of an on-site diagnostic PCR during pre-mating inspection in mares with the consequent segregation of EHV-3 positives, together with the application of a topical treatment in mares segregated, would minimize EHV-3 transmission, avoiding the infection of stallions, the possible progression to an outbreak, and, hence, the temporal disruption of mattings.
